# Physical activity in England: who is meeting the recommended level of participation through sports and exercise?

**DOI:** 10.1093/eurpub/cks127

**Published:** 2012-10-04

**Authors:** Nana Kwame Anokye, Subhash Pokhrel, Martin Buxton, Julia Fox-Rushby

**Affiliations:** Health Economics Research Group (HERG), Brunel University, Uxbridge, Middlesex, UK

## Abstract

**Background:** Little is known about the correlates of meeting recommended levels of participation in physical activity (PA) and how this understanding informs public health policies on behaviour change. **Objective:** To analyse who meets the recommended level of participation in PA in males and females separately by applying ‘process’ modelling frameworks (single vs. sequential 2-step process). **Methods:** Using the Health Survey for England 2006, (*n* = 14 142; ≥16 years), gender-specific regression models were estimated using bivariate probit with selectivity correction and single probit models. A ‘sequential, 2-step process’ modelled participation and meeting the recommended level separately, whereas the ‘single process’ considered both participation and level together. **Results:** In females, meeting the recommended level was associated with degree holders [Marginal effect (ME) = 0.013] and age (ME = −0.001), whereas in males, age was a significant correlate (ME = −0.003 to −0.004). The order of importance of correlates was similar across genders, with ethnicity being the most important correlate in both males (ME = −0.060) and females (ME = −0.133). In females, the ‘sequential, 2-step process’ performed better (ρ = −0.364, *P* < 0.001) than that in males (ρ = 0.154). **Conclusion:** The degree to which people undertake the recommended level of PA through vigorous activity varies between males and females, and the process that best predicts such decisions, i.e. whether it is a sequential, 2-step process or a single-step choice, is also different for males and females. Understanding this should help to identify subgroups that are less likely to meet the recommended level of PA (and hence more likely to benefit from any PA promotion intervention).

## Introduction

Although physical activity (PA) is a major preventative factor for disease conditions including coronary heart disease, cancer and stroke,^1^^,^^2^ the health gain is related to the level and intensity of activity.^3–8^ Therefore, both global and national public health policies are geared towards encouraging people to meet recommended levels of participation.^9^^,^^10^ However, Only 39% of men and 29% of women in England are ‘physically active’.^11^ A key challenge is therefore ‘how to encourage more people to become more active’.^9^ Understanding the correlates of PA could help to identify target areas (e.g. low income earners) for policy and lead to increasing the proportion of physically active individuals.

A ‘physically active’ adult is defined as someone who meets the following: either moderate intensity for at least 30 minutes on 5 days each week or vigorous intensity for at least 20 minutes on 3 days each week or equivalent combinations of moderate- and vigorous-intensive activities.^10^^,^^12^ Either recommended ‘dose’ corresponds to a weekly expenditure of 800–1200 calories, given body weight, and is seen as the minimum level required to gain health benefit.^13^

Little attention has been paid to the correlates of being ‘physically active’.^14–16^ The limited literature in England^17^^,^^18^ and elsewhere^14–16^ models whether individuals meet the recommended level of PA as a single decision process, using binary estimators that assumes individuals decide whether to undertake a level of activity that is sufficient to meet the recommended levels. It is, however, possible that meeting the recommended level may be a dual decision process involving a sequence of two separate, but connected, decisions. A person might first decide whether to participate in any PA, and then take a decision about the frequency, intensity and duration.^19–22^ As four studies^19–22^ have found an endogenous relationship between the dual decisions on participation and the frequency/duration of participation. Any attempt to model correlates needs to consider and correctly specify the dynamics of the decision process to avoid the risk of making incorrect policy recommendations.^20^ For example, modelling the dynamics of becoming ‘physically active’ as a single stage decision process is unlikely to encourage comprehensive policy leads ranging from discriminatory strategies to promote only uptake of PA and broader strategies that influence both uptake and level of uptake.

Whether a sequential 2-step process (hereafter referred to as ‘dual process’) modelling of this decision could provide more accurate policy leads than those from the existing literature is open to scrutiny. To our knowledge, only Eberth & Smith^20^ have modelled meeting the recommended level as a dual decision-making process. They investigated the impact of the number and intensity of activities on meeting the recommended level in Scotland. However, their analysis does not take into account the following three important aspects. First, it is not clear from their study how the correlates of meeting the recommended level are affected by different process modelling frameworks. Second, as gender has been shown to work differentially on the dynamics of PA behaviour,^21^^,^^23–25^ it is important that the analysis considers male and female samples separately, rather than pooling them together as was the case in their study. Although having gender as an explanatory variable in combined sample is useful in identifying which gender is likely to participate in PA (and undertake a level of activity that meets the recommended level), it does not show which process best predicts both decisions by gender and how correlates of participation differ across gender. Third, it is not clear whether their results will extend readily to an English population, as it is known that the Scottish population, on which their study was based, differs significantly from the English population in terms of demographics and uptake of PA.^26^

The aim of this article is therefore to analyse, using a nationally representative English sample, the decision to undertake a level of activity that is, in fact, sufficient to meet the recommended levels in PA in males and females separately by applying ‘process’ modelling frameworks (single vs. sequential, 2-step process).

## Methods

### Econometric specification

If undertaking a level of activity that is sufficient to meet the recommended level is considered as a single decision, a single probit regression model is used to estimate the correlates of that decision. However, if a dual decision process is considered, a two-equation model is required. The estimation of these two equations as separate discrete models is not appropriate because they are sequential choices that need to be modelled jointly, given the potential correlation between the error terms. In addition, the observed data for meeting the recommended level may not be random, as it is conditioned on participation in PA; hence, sample selection bias is likely. Failure to account for sample selection bias yields inconsistent estimates.^27^ We used a bivariate probit model with selectivity correction^28^ to estimate the correlates of participating in PA and meeting the recommended level, given participation.

In practice, the bivariate probit model with selectivity correction is estimated using first, a probit model of the probability that an individual participates in PA (Yp):
(1)
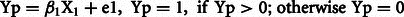

where β1, a vector of variables affecting the decision to participate in PA; e1, the error term.

And second, a probit model indicating that the individual meet the recommended level of participation (if Yp = 1) is estimated as:
(2)
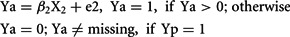

where β2, a vector of variables affecting the decision to undertake a level of activity that is sufficient to meet the recommended level; e2, the error term.

It is assumed that the two error terms for equations 1 and 2 are jointly normally distributed, and hence the selection model is estimated as:
(3)
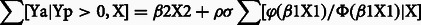

where Φ(.), the cumulative distribution of the standard normal distribution; φ (.), the corresponding density; σ, the variance of e_2__;_ ρ, the parameter of correlation between e_1_ and e_2_.

### Data

Data from the 2006 Health Survey for England, a cross-sectional survey that draws a nationally representative sample of 14 142 adults (≥16 years) in England, were used. Sampling was based on a multi-stage stratified random sampling approach. Data collection involved face-to-face interviews, self-completion, and clinical and physical measurements. Interviews were undertaken between January 2006 and May 2007 to account for seasonal variation. The main topics covered were: PA, general health, smoking and alcohol intake.

### Dependent variables

The decision to participate in PA is measured with a variable that indicates whether respondents had done any vigorous sports and exercise activities during the past 4 weeks. Box 1 gives details of the survey questions.

**Box 1 Health Survey for England (2006) questions on participation in PA**

Respondents were asked: ‘Can you tell me if you have done any activities on this card during the last four weeks that is since (date four weeks ago)? Include teaching, coaching, training and practice sessions’. The possible responses were ‘yes’ or ‘no’.

The list of activities included ‘swimming, cycling, workout at gym/exercise bike/weight training, aerobics/keep fit/gymnastics/dance for fitness, any other type of dancing, running/jogging, football/rugby, badminton/tennis, squash and exercises (e.g. press ups, sits ups)’.

Follow-up questions were administered to probe further for other activities the respondents may have done, and also collect data on the intensity, frequency and duration of days of participation.

Source: Health Survey for England 2006.

The indicator of meeting the recommended level was based on the ‘number of days of vigorous sports done during the last four weeks at the recommended duration’ (at least 20 minutes). It takes the value of one if at least ‘12 days of vigorous sports was done during the last four weeks at the recommended duration’ and zero otherwise.

### Explanatory variables

Our model includes economic, socio-demographic, health and other variables that in previous research^21–25^ had been shown to correlate with PA. Table 1 shows these variables and their distributions based on means (standard deviation) and proportions. Income is specified as ‘equivalized income’, as that reflects the ‘real’ income of the household by adjusting for its size and composition because standard of living varies in households with same total income but different composition.^29^

**Table 1:** Descriptive statistics of variables (adjusted for missing observations)

Variables^a^	Observations	Mean (SD)/%
Dependent		
Participate in PA		
Yes	6248	44.2
Missing	10	0.07
Meeting recommended level		
Yes	1343	9.5
Explanatory		
Educational qualification		
Yes	2711	19.2
Missing	48	0.3
Employed		
Yes	7642	54.0
Missing	40	0.3
Age	14 142	49.3 (18.6)
Number of adults in household	14 142	2.2 (0.92)
Access to vehicle	11 532	81.5
Yes	11 466	81.1
Missing	3	0.01
Ethnicity		
White	12 834	89.1
Mixed	123	1.0
Asian	831	5.9
Black	395	2.8
Chinese	158	1.1
Missing	35	0.01
Gender		
Male	6324	44.7
Female	7818	55.3
Marital status		
Other	2872	20.3
Married	7709	54.5
Single	3558	25.2
Missing	3	0.01
Income	14 142	28358.6 (23751.9)
Missing^b^	2792	19.7
Working hours		
Fulltime	9412	66.6
Part time	3923	27.7
Missing	807	5.7
Number of children	14 142	0.5 (0.90)
Drinkers		
Yes	11 295	79.9
Missing	87	0.6
Smokers		
Yes	3101	21.9
Missing	107	0.8
Voluntary activities		
Yes	1539	10.9
Missing	1602	11.3
Health status		
Good health	10 464	73.1
Fair health	2650	18.7
Poor health	1025	7.3
Missing	3	0.01
Urban residence		
Yes	10 979	77.6
Seasonal effect		
Summer	3224	22.8
Spring	3535	25
Autumn	3592	25.4
Winter	3790	26.8
Region of residence		
North east	738	5.2
North west	1918	13.6
Yorkshire	1429	10.1
East Midlands	1238	8.8
West Midlands	1498	10.6
East	1573	11.1
London	2011	14.2
South west	1440	10.2
South east	2297	16.2
Obese (BMI 30+ kg/m^2^)		
Yes	3010	21.3
Missing	2115	15.0

SD, standard deviation.

a: The observations for ‘no’ category for variables with yes/no categories can be derived by finding the difference between 100 and the sum of the observations for categories presented on table.

b: Mean (SD) unadjusted for missing observations is 29112.2 (2569.4).

### Instrumental variables

To guarantee unique estimates for the two equations, the first probit model is identified via exclusion criteria (at least one or more explanatory variables in that model should not enter the second probit model).^30^ It is, however, often difficult to select the instruments for the exclusion criteria.^30^^,^^31^ Therefore, evidence from the PA literature was first sought. Humphreys and Ruseski^21^ using a US sample suggests ‘number of children’, ‘region of residence’ and ‘health status’ as good instruments because they are correlates of participating in PA, but not the level of participation. Although the instruments were used in a PA context, they may not be effective in this present study, as behaviour patterns differ across countries. As the only study in this respect, the evidence base was considered not strong. Therefore, we identified additional set of instruments specific to our sample (using bivariate regressions between the dependent and explanatory variables). Estimates of selection bias based on either set of instrument were compared. Although we recognize that the instruments used in our study may not be generalizable across other samples, they were based on a mix of theoretical and empirical findings.^21^

### Selection bias

Selection bias is suggested if the correlation coefficient (ρ) between the error terms of the two equations of the bivariate probit model with selectivity correction is found to be statistically significant.^30^ The likelihood ratio test was used to investigate this.^32^

### Tests of models

To test which of the decision processes best describes the decision to undertake a level of activity that is sufficient to meet the recommended level, the Hausman specification test was used to check which estimator produced better consistent and efficient estimates.^33^ Given that the single decision model does not include a variable on ‘participate or not’, it is not possible to simply compare the height of its coefficients with that of the dual decision model because both models are subject to varied normalization processes. Hence, a differential normalization is applied by equating a parameter to one in both models to allow the test of equality.

### Analysis

We used the chi-square and Fischer’s exact tests to examine whether missing data occurred completely at random.^34^ If at random, regression-based imputation method was used to replace missing values of continuous variables, and a dummy variable specifying item non-response was added. For categorical variables, item non-response was included in the omitted category, and a dummy variable for item non-response was created.^35^

The models were estimated with sampling weights that were calculated as the inverse of the probability of being a respondent in a household multiplied by the household weight, which accounts for non-responding households.^13^ MEs were computed for each variable. The threshold for statistical significance was set at ≤10% in all analyses because of the exploratory nature of the study. Analyses were undertaken using Stata 10.

## Results

Table 1 shows summary statistics for the variables. The sample was predominately White (89%) and had a mean age of 49 (SD 19) years. Most were female (55%), married and living with their partners (55%) and reported good health status (73%). Few (21%) were obese or smokers (22%), although the majority were ‘drinkers’ (80%). In all, 44% stated they had participated in PA, and 10% were judged to meet the recommended level, based on their self-reported data. These statistics are comparable with English population estimates.^36^^,^^37^

The ‘decision to participate in PA’ had 10 missing observations, whereas ‘meeting the recommended level given participation’ had none. All explanatory variables (except region of residence, age, gender, urban residence, number of children, number of adults and seasonal effect) had missing observations. ‘Obese’ had the highest number of missing observations (*n* = 2115), whereas ‘marital status’ and ‘health status’ had the lowest (*n* = 3). The proportion of PA participants with missing values for explanatory variables were significantly different from ‘non-participants’, (except ‘marital status’, ‘working hours’, ‘drinking status’, ‘smoking status’ and ‘access to vehicle’).

### Regression models

#### Instrumental variables

The bivariate regression analyses showed that ‘number of children’ is correlated with the decision to participate in PA, but not to undertake a level of activity that is sufficient to meet the recommended level and hence could be an extra instrumental variable.

Similar results were found when selectivity bias in the models were checked using the two different exclusion criteria [(i) ‘number of children’—via empirical evidence in the data set, and (ii) ‘number of children’, ‘region of residence’ and ‘health status’—via evidence from literature]. In both cases, there was no difference in the statistical significance of the correlation coefficient between the error terms of the selection and outcome equations. Therefore, the instrumental variables in the literature^21^ were chosen.

#### Selection bias

Tables 2 and 3 show coefficients and MEs of the reduced models for female and male samples, respectively. Estimates are provided separately for dual decision (bivariate probit with selectivity correction) and single decision models (ordinary probit). In the female sample, the correlation coefficient (ρ = −0.364) was statistically significant at 1%, indicating that sample selection bias did occur in that sample, and thus adjusting for the decision to participate was important to identify the correlates of meeting the recommended level. This is confirmed by the likelihood ratio test that showed the decision to participate was correlated with meeting the recommended level (chi^2 ^= 10.39; *P* = 0.001). No such correlation was observed in the sample of males (ρ = 0.154).

**Table 2:** Estimation results of bivariate probit model with selectivity correction and ordinary probit: female sample

Explanatory variables	Bivariate probit with selectivity correction				Ordinary probit	
	Participate or not		Meeting the recommended level		Meeting the recommended level	
	Coef^a^.	ME	Coef^a^.	ME	Coef^a^.	ME
Degree qualification	0.196***	0.077			0.140**	0.013
Employed			0.198***	0.054	0.217***	0.019
Age	−0.018***	−0.007			−0.010**	−0.001
Ethnicity^b^						
Mixed	0.105	0.046	−0.179	−0.045		
Asian	−0.422***	−0.155	0.422***	0.133		
Black	−0.046	−0.016	0.002	0.001		
Chinese	0.036	0.015	−0.246	−0.060		
Income			0.000**	0.000	0.000**	0.000
No. of children	−0.002	−0.001				
Drinkers	0.314***	0.123				
Smokers	−0.195***	−0.075				
Health status^c^						
Good health	0.487***	0.181			0.570**	0.039
Fair health	0.280***	0.111			0.231	0.023
Obese	−0.107**	−0.045	−0.163**	−0.042	−0.160	−0.013
Voluntary activity	0.114**	0.046				
Urban residence			0.170**	0.044		
Seasonal effect^d^						
Summer	0.203***	0.081	0.154**	0.043	0.254***	0.025
Spring	0.061	0.024	0.017	0.005	0.065	0.006
Autumn	0.059	0.024	−0.002	−0.001	0.032	0.003
Region of residence^e^						
North east	−0.123	−0.050				
North west	−0.214***	−0.089				
Yorkshire	−0.133**	−0.054				
East Midlands	−0.037	−0.014				
West Midlands	−0.095	−0.040				
East	0.010	0.000				
London	−0.189***	−0.081				
South west	−0.018	−0.011				
Constant	−0.201*		−1.328***		−2.272***	
Observations	7818		3349		7818	
Rho	−0.364		−0.364			
	*P = *0.001		*P = *0.001			

Coef, Coefficients; ME, Marginal effects.

a: The estimated parameters and asterisks show significance level of 1% (***), 5% (**) and 10% (*).

b: Reference category: white.

c: Reference category: poor health.

d: Reference category: winter.

e: Reference category: south east.

Hausman specification test (null hypothesis: difference in coefficients in two models is not systematic): Chi^2^ (7) = 107.32; *P = *0.001.

**Table 3:** Estimation results of bivariate probit model with selectivity correction and ordinary probit: male sample

Explanatory variables	Bivariate probit with selectivity correction				Ordinary probit	
	Participate or not		Meeting the recommended level		Meeting the recommended level	
	Coef^a^.	ME	Coef^a^.	ME	Coef^a^.	ME
Degree qualification	0.159***	0.064				
Employed	−0.081*	−0.032				
Age	−0.022***	−0.009	−0.017***	−0.004	−0.021***	−0.003
Access to vehicle			−0.184**	−0.047	−0.148**	−0.026
Ethnicity^b^						
Mixed			0.130	0.033	0.016	0.003
Asian			−0.080	−0.018	−0.100	−0.016
Black			−0.042	−0.010	−0.104	−0.016
Chinese			−0.611**	−0.104	−0.519**	−0.060
Marital status^c^						
Other	0.028	0.012				
Married (living)	−0.106**	−0.041				
Income	0.000***	0.000	0.000***	0.000	0.000***	0.000
Full time work	−0.139**	−0.055				
No. of children	0.050	0.019***				
Drinkers	0.135**	0.052				
Smokers	−0.215***	−0.085	−0.302***	−0.067	−0.304***	−0.045
Voluntary activity						
Health status^d^						
Good health	0.717***	0.276			0.339**	0.050
Fair health	0.431***	0.163			−0.041	−0.022
Obese	−0.131***	−0.051	−0.301***	−0.065	−0.274***	−0.041
Seasonal effect ^e^						
Summer	0.309***	0.121	0.070	0.017	0.143**	0.025
Spring	0.131**	0.052	0.101	0.025	0.131**	0.023
Autumn	0.135***	0.053	0.177**	0.044	0.172**	0.030
Region of residence^f^						
North east	−0.405***	−0.159				
North west	−0.239***	−0.095				
Yorkshire	−0.179**	−0.073				
East Midlands	−0.128*	−0.053				
West Midlands	−0.132*	−0.054				
East	−0.074	−0.033				
London	−0.268***	−0.107				
South west	−0.102	−0.044				
Constant	0.140		−0.263			
Observations	6324		3425		6324	
Rho	0.154		0.154			
	*P = *0.524		*P = *0.524			

Coef, Coefficients; ME, Marginal effects.

a: The estimated parameters and asterisks show significance level of 1% (***), 5% (**) and 10% (*).

b: Reference category: white.

c: Reference category: single.

d: Reference category: poor health.

e: Reference category: winter.

f: Reference category: south east.

Hausman specification test (null hypothesis: difference in coefficients in two models is not systematic): Chi^2^ (7) = 10.13; *P = *0.60.

#### Dual-stage decision process

Among females, health status was the most important explanatory variable of the ‘decision to participate’, as women with good health were 18% more likely to participate in PA compared with those with poor health (table 2). Compared with Whites, Asians (excluding Chinese; hereafter this specification applies) were 18% less likely to participate in PA. Degree holders, drinkers, voluntary activity practitioners and summer season were positively associated with participation, whereas older people, obese, smokers and London, North West and Yorkshire residents were negatively associated. Similar results were found in males but with minor exceptions (table 3). For example, although high income earners and residents of households with more children were more likely to participate in PA, the employed, full-time workers and married men were less likely.

Results on meeting the recommended level were different, as some correlates of the decision to participate in PA were not significant here and vice versa. For instance, age and educational qualification of females explained their participation in PA but not meeting the recommended level. Conversely, ethnicity was correlated with males meeting the recommended level but not the initial decision to participate.

Relatively few variables emerged as correlates of meeting the recommended level in both genders. Ethnicity was the most important explanator in females, as Asian females were 13% more likely to meet the recommended level (table 2). Other positive correlates in females were income, urban residence and summer season, whereas obesity was a negative correlate. For males, ethnicity was the most important explanator with Chinese men 16% less likely to meet the recommended level. Older males, smokers, obese people and access to a vehicle were also negatively associated with meeting the recommended level. Income and autumn season were, however, positive correlates.

#### Single-stage decision process

Although correlates of meeting the recommended level were slightly different between the single and dual-stage processes among females, it was similar among males. For example, in females, degree holders (ME = 0.013) and older people (ME = −0.001) were correlates only when meeting the recommended level was modelled as a single-stage process. Conversely, all the other correlates in the dual-stage process were found in single stage with the exception of ethnicity. The order of importance of the correlates was similar across both decision processes.

#### Tests of models

The Hausman specification test suggested that among females, the parameters of the dual-decision model were systematically different from those of the single decision model (Chi^2 ^= 107.32; *P = *0.001) and produced better consistent and efficient estimates with the former model generating lower coefficients than the latter (table 2). Both models, however, yielded similar coefficients in males.

## Discussion

In this article, an effort was made to test the construct that meeting the recommended level PA is best modelled as a dual-decision process. In our sample, it was evident in females, but not in males. The findings should, however, be interpreted with caution because meeting the recommended level was specified only in terms of vigorous intensity PA owing to data limitations. Therefore, it is still unknown whether and how these findings relate to other types of PA (e.g. moderate intensity PA). This is important to know because the dynamics of PA behaviour tend to vary depending on the type of PA in question.^38^ Further research in this area will help us improve our current understanding.

Findings could be questioned in terms of whether the choice of instrumental variables introduces bias. One of the instruments (i.e. number of children) was not a correlate of participation among females, thereby raising questions about whether the systems of equations are properly identified. However, findings on selection bias were consistent across instruments that excluded (or included) this variable. In addition, a system of equations is appropriately identified even with one instrument.^39^

Also of potential concern is the low impact of income. This could be debated on grounds that variables such as age, number of children and adults in household may have been highly correlated with income, and hence minimized its influence because the income variable was derived by adjusting household income using those variables. However, the robustness of the finding on income is justified owing to a number of reasons. First, the collinear levels of these variables including income were within acceptable levels, with average variance inflation and tolerance indicators of 1.6 and 0.6, respectively. Second, the magnitude of the effect of income was consistent in reduced models, which excluded those variables.

Using subjective measures of PA, regardless of validity and reliability tests, may be fraught with overestimation/problems with recall.^40^ Logistical constraints precluded the use of objective measures. Nonetheless, the use of sports and exercise to indicate PA here is likely to offer better recall, as these activities are usually undertaken in a premeditated way.^11^

The key message of this study is that the degree to which people undertake the recommended level of PA through vigorous activity varies between males and females, and the process that best predicts such decisions, i.e. whether it is a sequential, 2-step process or a single-step choice is also different for males and females. This has a number of policy implications. First, such understanding helps to identify subgroups that are less likely to meet the recommended level of PA through vigorous activity (and hence more likely to benefit from any PA promotion intervention) by providing an unbiased effect size. Ignoring the dual decision-making process could lead to incorrect policy recommendations, particularly for females. Had this not been the case, the residents outside urban areas who might be an important policy target for improving adherence to meeting the recommended level would not have been identified when the single-decision process is assumed. Conversely, older people would be wrongly identified as a target group in the context of single-decision process. Second, strategies for encouraging people to participate in PA may not be effective in encouraging those already doing PA to do more. This is hinted at by the finding that some factors may influence take-up but not meeting the recommended level given participation. For example, devising a policy to target older and less-educated people may increase uptake in PA, but have no impact on whether the recommended level of PA is met. On the other hand, interventions that target obese people are likely to influence both sets of decisions. This offers policy options ranging from discriminatory strategies to promote only take-up and broader strategies that influence both uptake and level of uptake of vigorous activity. Finally, we recognize that single and dual decision-making processes should be examined in the context of achieving recommended levels of PA through moderate exercise and, on the basis of these findings, we recommend that such analysis also considers males and females separately, as decision making strategies could differ.
